# Embryo cell allocation patterns are not altered by biopsy but can be linked with further development

**DOI:** 10.1530/REP-17-0514

**Published:** 2017-12-16

**Authors:** L P Sepulveda-Rincon, N Islam, P Marsters, B K Campbell, N Beaujean, W E Maalouf

**Affiliations:** 1 Division of Child HealthObstetrics and Gynaecology, School of Medicine, University of Nottingham, Nottingham, UK; 2 Univ LyonUniversité Claude Bernard Lyon 1, Inserm, INRA, Stem Cell and Brain Research Institute U1208, USC1361, 69500 Bron, France

## Abstract

It has been suggested that first embryo cleavage can be related with the embryonic–abembryonic axis at blastocyst stage in mice. Thus, cells of the 2-cell embryo might be already biased to form the inner cell mass or trophectoderm. This study was conducted to observe the possible effects of embryo biopsy on cell allocation patterns during embryo preimplantation in two different mouse strains and the effects of these patterns on further development. First, one blastomere of the 2-cell embryo was injected with a lipophilic tracer and cell allocation patterns were observed at blastocyst stage. Blastocysts were classified into orthogonal, deviant or random pattern. For the first experiment, embryos were biopsied at 8-cell stage and total cell counts (TCC) were annotated. Furthermore, non-biopsied blastocysts were transferred into foster mothers. Then, pups and their organs were weighed two weeks after birth. Random pattern was significantly recurrent (≈60%), against orthogonal (<22%) and deviant (<22%) patterns among groups. These patterns were not affected by biopsy procedure. However, TCC on deviant embryos were reduced after biopsy. Moreover, no differences were found between patterns for implantation rates, litter size, live offspring and organ weights (lungs, liver, pancreas and spleen). However, deviant pups presented heavier hearts and orthogonal pups presented lighter kidneys among the group. In conclusion, these results suggest that single blastomere removal does not disturb cell allocation patterns during pre-implantation. Nonetheless, the results suggest that embryos following different cell allocation patterns present different coping mechanisms against *in vitro* manipulations and further development might be altered.

## Introduction

Assisted reproductive technologies (ARTs) have been clinically used for more than three decades and the success rates still remain relatively low, with a probability to take home a baby after an IVF cycle of around 33% in women younger than 35 years old (Botros *et al.* 2008) and no more than 23% for women of older age groups (HFEA 2013). Several factors have been related with pregnancy outcomes after ARTs as gametes/embryos origin and state (autologous or donation and fresh or thawed), female stimulation treatment, patient age, number of previous treatments and day of embryo transfer (D3 or D5) to mention the most common ones (Meseguer *et al.* 2012). Nevertheless, embryo implantation failure remains the main cause of the low success rates on ARTs (Hesters *et al.* 2008). Most of embryo implantation failures or pregnancy arrests are caused by embryo chromosomal or genetic abnormalities (Simpson 2012); therefore, pre-implantation genetic screening (PGS) or diagnosis (PGD) before embryo transfer is suggested for those cases. The main objectives of PGD are to improve birth rates in those patients presenting any kind of genetic disease or disorder and to reduce spontaneous abortions (Munné *et al.* 2010). Embryo pre-implantation genetic diagnosis/screening (PGD/S) is becoming increasingly applied in fertility clinics. Even though trophectoderm biopsy is currently becoming the preferred method, blastomere biopsy on day 3 remains the most common technique for obtaining the biological material according to the latest ESHRE (European Society for Human Reproduction and Embryology) consortium (De Rycke *et al.* 2015). So far, more than ten thousand babies have been born after a PGD/S cycle (Peyvandi *et al.* 2011). Therefore, further research on the potential effect and safety of embryo biopsy on embryo development is needed.

Until recent years, it was believed that during the first round of cleavage, the cells in the mammalian embryo were identical and had the same potential to become ICM or TE. However, the literature reports a theory called the pre-patterning or biased theory, which refers to the different potential of the twin blastomeres at the two-cell stage embryo to become ICM or TE (Piotrowska *et al.* 2001, Piotrowska & Zernicka-Goetz 2002, Fujimori *et al.* 2003, Gardner 2007, Torres-Padilla *et al.* 2007, Bischoff *et al.* 2008, Katayama & Roberts 2010, Liu *et al.* 2012). Nevertheles, this theory has been debated suggesting that totipotency within the embryo blastomeres is maintained up to the 8-cell stage (Alarcón & Marikawa 2003, Motosugi *et al.* 2005, Waksmundzka *et al.* 2006, Kurotaki *et al.* 2007, Alarcón & Marikawa 2008, González *et al.* 2011, Wennekamp *et al.* 2013). It is still unclear if mammalian embryos are pre-patterned or the presence of stochastic development is just a reflex of the great plasticity of mammalian embryos. Research performed on cell allocation patterns suggest that it might be a common characteristic during pre-implantation embryo development of different mammalian species (Park *et al.* 2009, Hosseini *et al.* 2016, Sepulveda-Rincon *et al.* 2016). However, there is a lack of evidence on the mechanism(s) leading to these cell allocation patterns. In this work, the murine model is used to investigate the cell allocation pattern incidence among two different mouse strains and the effects of embryo blastomere removal at the cleavage stage on these patterns. Additionally, we investigated the effect of cell allocation patterns on further embryo development and organ morphometry using the murine model because of its short gestational period.

## Materials and methods

All experiments were performed according to the Animals Scientific Procedures Act, 1986 under the Home Office licence 40/3480 and with the approval of the Bio Support Unit at the University of Nottingham. Two different experiments were performed during the present study. First, the effects of single blastomere removal at the 8-cell stage on cell allocation patterns were studied using two different mouse strains. Second, the effects of cell allocation patterns on further embryo development were addressed.

### Mouse embryo production

For biopsy experiments, B6CBAF1 × B6CBAF1 fresh embryos and B6C3F1 × B6D2F1 frozen/thawed embryos were used. First, 5- to –7-week-old B6CBAF1 females (Charles River) were superovulated with one peritoneal injection of 5 IU pregnant mare serum gonadotropin (PMSG, Intervet, Buckinghamshire, UK) followed by one peritoneal injection of 5 IU human chorionic gonadotropin (hCG, Intervet) after 48 h. After hCG injection, females were placed with males of the same strain and vaginal plugs were checked the following morning. Females were killed by cervical dislocation, and embryos were collected at one-cell stage at 22–24 h post hCG. Embryos were collected in EmbryoMax Hepes medium (Merck Millipore), previously equilibrated at 37°C. In order to compare the obtained results, a different mouse strain was used, B6C3F1 × B6D2F1. One cell frozen B6C3F1 × B6D2F1 mouse embryos (Embryotech Laboratories, Inc., Haverhill, MA, USA) were thawed in EmbryoMax Hepes medium (Merck Millipore) according to supplier’s instructions. After embryo thawing, embryos were cultured in KSOM medium (Merck Millipore) under mineral oil (Merck Millipore) at 37°C and 5% CO_2_. For the second part of the study, the embryo transfer experiment, only frozen/thawed B6C3F1 × B6D2F1 embryos were used.

### Non-invasive cell tracing method

As previously reported (Sepulveda-Rincon *et al.* 2016), the lipophilic tracer CM-DiI (Chloromethyl DiI, Molecular probes) was dissolved in olive oil at a final concentration of 2 mg/mL. Prior to labelling, the FemtoTip II (Eppendorf, Hamburg, Germany) was backfilled with the dye. Mouse embryos at 2-cell stage were placed in HEPES buffered medium at 37°C during the micromanipulation. The injections were performed on an inverted microscope (Leica DMI3000 B) using Eppendorf TransferMan NK 2 micromanipulators with a coupled Eppendorf FemtoJet microinjector. The micropipette was pushed through the zona pellucida and pressed against one of the blastomere membranes, and then a microdrop (≈5 pL) was deposited.

### Cleavage stage embryo biopsy

Mouse embryos were assessed at 68–72 h post hCG or 43–45 h post thawing. Then, 6- to 8-cell embryos were randomised for biopsy. The selected embryos were transferred to a pre-warmed 60 mm ICSI dish (BD Falcon) containing 10 µL drops of G-PGD medium (Vitrolife) under mineral oil (Merck Millipore). Briefly, embryo biopsy was performed using a 40× XYclone laser objective (Hamilton Thorne Biosciences, Beverly, MA, USA) mounted on a Leica DMI3000 B inverted microscope. A single blastomere was randomly removed and the resulting biopsied embryos were further cultured in culture medium until the blastocyst stage under culture conditions. For morphokinetic analysis, embryos were cultured in a time lapse imaging incubator (EmbryoScope, Vitrolife, Denmark).

### Blastocyst assessment

#### Cell allocation patterns

Blastocysts were scored for the different pattern categories as described previously (Sepulveda-Rincon *et al.* 2016). Briefly, blastocysts were placed in 5 µL EmbryoMax Hepes (Merck Millipore) medium drops on 60 mm petri dish and on a Leica DMI3000 B inverted microscope, coupled with red fluorescent filters corresponding to maximum excitation/emission wavelengths of 553 nm and 570 nm respectively. For better visualisation, embryos were rotated in order to place the blastocoel cavity floor and the boundary line between the fluorescent and non-fluorescent cells in the same focal plane. Then, embryos were classified into three categories: orthogonal, if the Em-Ab axis was orthogonal ±30 to the boundary line between stained and non-stained cells; deviant, if it was ±30 to the Em-Ab axis and random, if stained cells were intermingled with non-stained cells and more than 2 clusters of cells were observed.

#### Blastocyst total cell count

Blastocysts were permeabilised in 0.1% Triton-100X/PBS during two minutes at room temperature and then rinsed twice in phosphate buffered saline (PBS). Finally, embryos were mounted on a SuperFrost microscope slide using Vectashield-DAPI as mounting medium and nuclear staining. Total cell count was effectuated on a Nikon Eclipse Ti 90× microscope, along with a Hamamatsu digital camera (C4742-80-12AG) and a fluorescent filter corresponding to the excitation 350 nm and 470 nm of emission for DAPI.

#### Blastocyst expansion grading

Blastocysts for embryo transfer were graded according to their blastocoel expansion according to the Gardner and Schoolcraft grading system. Blastocysts in which the blastocoel cavity was half or it completely fills the embryo were classified as Grade ≤3; Grade 4 blastocysts were those which the blastocoel cavity fully fills the embryo and this was bigger than the original volume and zona pellucida thinning was observed; Grade 5 blastocysts—some cells are herniating through the zona pellucida.

### Non-surgical embryo transfer

Nine- to eleven-week-old CD1 female mice were placed with >10 weeks old vasectomised CD1 males. Then, 2.5 days post coitus (dpc) pseudo-pregnant females were used as surrogates for embryo transfer. The protocol suggested on the non-surgical embryo transfer (NSET) device (ParaTechs, Lexington, KT, USA) was followed. Briefly, after cell allocation pattern classification, embryos were transferred by groups to 15 µL drops of KSOM previously equilibrated at 37°C. Then, 13–18 blastocysts were loaded into the NSET device, and it was inserted into the mouse cervix where embryos were released. After embryo transfer, foster females were caged individually and pregnancy was visually assessed on day 14 after embryo transfer.

### Offspring assessment

Pups were weighed and sexed at 1 week after birth. Furthermore, pups were culled 2 weeks after birth by cervical dislocation, weighed and dissected. Different organs (spleen, pancreas, kidneys, liver, lungs and heart) were carefully dissected and weighed immediately after harvesting. Relative organ weights were calculated dividing organ weight by total body weight. Foster mothers were also killed by cervical dislocation. Then, uteri were harvested and implantation sites were noted.

### Statistical analysis

Data distribution was checked for normality by Shapiro–Wilk test and using SPSS, v23 (IBM Software Services). Presented values are mean value ± standard error of mean. For values reporting cell allocation pattern incidence, s.e.m. was calculated based on the number of repetitions of the experiment, whereas for values reporting TCC, s.e.m. was calculated based on total number of embryos analysed. Statistical significance was set at *P* < 0.05 for all analyses. Blastocysts incidence rates within the different cell allocation patterns were compared using ANOVA test for single or two factor as needed. *Post hoc* Bonferroni test was used when adequate. Non-parametric test, Kruskal–Wallis, was used to determine any differences among groups for pregnancy, pregnancy loss, mean live birth, live offspring rates as well as litter size comparison. Also, non-parametric test Kruskal–Wallis was used to determine any differences on relative organ weights among groups; when adequate Mann–Whitney *U* test was used to compare groups against each other.

## Results

### Cleavage stage embryo biopsy does not disturb cell allocation patterns

**Figure 1:**
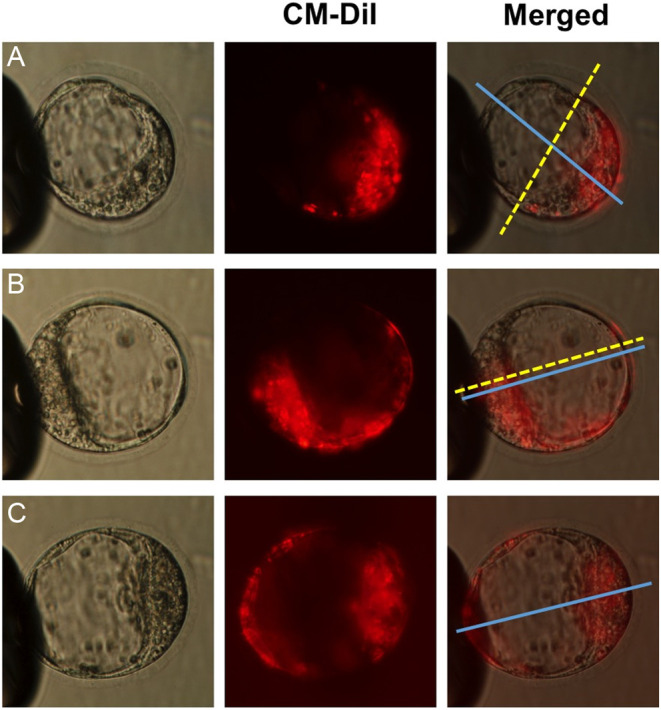
Mouse embryo cell allocation patterns at blastocyst stage. (A) Orthogonal embryos, the boundary (doted yellow line) of the stained and non-stained cells is orthogonal to the embryonic-abembryonic (Em-Ab) axis (blue line); (B) Deviant embryos, the boundary of stained and non-stained cells is parallel to the Em-Ab axis; and (C) Random embryos, stained and non-stained cells are randomly distributed and no boundary line between them can be seen.

**Figure 2:**
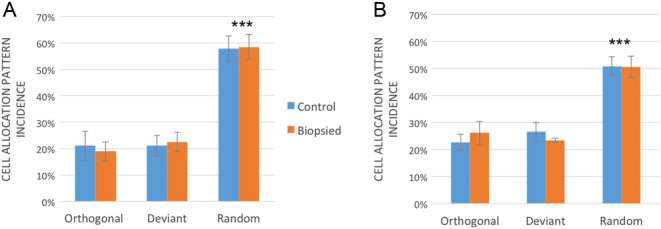
Incidence of different cell allocation patterns across control and biopsied mouse embryos. (A) Cell allocation pattern incidence in B6CBAF1 × B6CBAF1 blastocysts. (B) Cell allocation pattern incidence in B6C3F1 × B6D2F1 blastocysts. ***Denotes statistical difference *P* < 0.001.

A total of 287 B6CBAF1 × B6CBAF1 blastocysts in the control group and 214 blastocysts in the biopsied group were successfully classified according to their cell allocation pattern as previously reported on (Sepulveda-Rincon *et al.* 2016) (Fig. 1). Orthogonal embryos represented 21 ± 5.4% and 19 ± 3.6% of the blastocysts within the control and the biopsied group respectively. The deviant group represented 21 ± 3.9% and 23 ± 3.6% and the random group 58 ± 4.8% and 59 ± 4.6% for the control and biopsied group respectively (Fig. 2A). Univariate two-way ANOVA showed no significant differences between treatment: control and biopsied groups (*P* = 0.973). However, there is a significant difference on the incidence of the cell allocation patterns (*P* < 0.001). *Post hoc* Bonferroni test revealed that this difference is mainly attributed to the difference between the incidence of the random group compared with the orthogonal (*P* < 0.001) and deviant (*P* < 0.001). Morphokinetic analysis after blastomere removal (Supplementary Table 1, see section on supplementary data given at the end of this article) revealed that there are no differences between patterns among the control group (*P* < 0.05); however, this is not the case among the biopsied group (*P* = 0.012). Orthogonal embryos spend longer time on the interval from 8-cell stage to 9-cell stage (*P* = 0.011) when compared with deviant embryos. Also, orthogonal embryos spend longer time between compaction and the start of cavitation when compared with deviant (*P* = 0.003) and random embryos (*P* = 0.012).

In order to address if the biopsy procedure has an effect on the size of the embryo at blastocysts stage, total cell counts (TCC) of blastocysts were calculated. Embryos from the orthogonal group presented a TCC of 45.8 ± 0.2 cells and 41.2 ± 0.5 cells within the control and biopsied group respectively. Deviant embryos presented a TCC of 50.4 ± 0.6 and 42.0 ± 0.4 for control and biopsied group, while random embryos presented a TCC of 47.9 ± 0.2 and 43.5 ± 0.2. Univariate test showed no significant difference on the TCC among the three patterns (*P* = 0.490). Still, there was a significant difference on the TCC between the control and the non-biopsied groups (*P* = 0.002). *Post hoc* Bonferroni test suggested that this difference is mainly attributed to the effect of biopsy on the TCC of the deviant group (*P* = 0.028).

Similar results were obtained when repeating the above experiment on B6C3F1 × B6D2F1 frozen/thawed embryos. A total of 69 blastocysts in the control group and 82 blastocysts in the biopsied group were classified where 23 ± 2.9% and 26 ± 4.2% were orthogonal in the control and biopsied groups respectively, 26 ± 3.4% and 23 ± 0.7% were deviant, and 51 ± 3.5% and 50 ± 3.9% were random (Fig. 2B). No significant differences were found on the incidence of the different cell allocation patterns between treatment groups (*P* > 0.05). However, random embryos remained more predominant (*P* < 0.001) when compared with orthogonal and deviant embryos in both treatment groups. Morphokinetic analysis did not show any difference among cell allocation patterns in control and biopsied groups (*P* < 0.05) and even though a tendency of orthogonal embryos spending longer time between the 8-cell stage to the 9-cell stage, significance was not reached (*P* = 0.056, Supplementary Table 1). TCC at blastocyst stage were similar among cell allocation patterns in the control group (*P* = 0.683) or the biopsied group (*P* = 0.932). Again, significant difference was found on the TCC between control and biopsied group (*P* = 0.002), and it was attributed to the decreas of TCC of deviant embryos after biopsy (*P* = 0.030). Orthogonal embryos presented 49.8 ± 2.8 cells and 45.5 ± 1.4 cells for control and biopsied groups respectively, deviant embryos presented 52.1 ± 2.6 cells and 45.6 ± 1.5 cells and random embryos presented 50.0 ± 1.9 cells and 46.3 ± 1.2 cells respectively.

### Body weight and organ development might be affected by cell allocation patterns during preimplantation embryo development

As cell allocation patterns were not disturbed by cleavage stage embryo biopsy, further analysis on the effect of these patterns without the biopsy procedure was investigated. During three repetitions of the experiment, a total of 323 blastocysts were successfully classified into orthogonal, deviant or random accordingly to their cell allocation pattern with an incidence of 27.0 ± 4.7, 28.3 ± 5.2 and 52.3 ± 7.2% respectively.

In order to observe if there was the same distribution on blastocysts size within groups, blastocyst expansion grades were noted. Expansion grades were similar between orthogonal (*n* = 83), deviant (*n* = 85) and random (*n* = 157) groups (*P* = 0.717 for grade ≤3, *P* = 0.917 for grade 4 and *P* = 0.734 for grade 5 among groups by ANOVA test). The majority of the blastocysts were graded as grade 4; 57.8% for orthogonal, 60% for deviant and 57.3% for random groups. Smaller proportions of blastocysts grade 5 and ≤3 were found within the groups; 21.7% and 20.5% for orthogonal, 23.5% and 16.5% for deviant, 24.9% and 17.8% for random groups respectively.

Seventeen 2.5 dpc pseudo-pregnant females were used to transfer a total of 275 blastocysts. Due to the higher incidence of embryos classified as random, more foster mothers were used in this group. Nevertheless, the transferred blastocyst number within the three repetitions of the experiment remained constant within the three groups (18, 13 and 16 blastocysts per female for the first, second and third repetition respectively). No significant differences were found between groups for pregnancy rates, implantation rates, pregnancy loss rate, litter size or live offspring rates (Table 1) (*P* > 0.05 by Kruskal–Wallis test).

**Table 1:** Non-surgical embryo transfer results in mice.

**Embryo group**	**Orthogonal**	**Deviant**	**Random**
Transferred blastocysts	76	76	123
Surrogates	5	5	7
Pregnancy rate	66.6%	83.3%	100.0%
Implantation rate	42.3%	39.9%	50.7%
Pregnancy loss rate	49.6%	79.1%	55.9%
Total pups (litters)	12 (3)	8 (2)	25 (6)
Mean litter (s.d.)	4.0 (2.6)	4.0 (1.4)	4.1 (2.2)
Live offspring rate	14.7%	11.4%	22.9%

Total numbers and different rates (percentages) among groups are not significantly different (*P* > 0.05 by Kruskal–Wallis test).

s.d., standard deviation.

Significant difference was found (*P* = 0.028) among groups when 7-day-old pups weights were compared. Mann–Whitney *U* test revealed that pups from deviant group were significantly lighter (5.60 ± 0.3 g) when compared with pups from random group (*P* = 0.015, 6.55 ± 0.1 g), but not when compared to orthogonal group (*P* = 0.230, 6.28 ± 0.3 g). Fourteen-day-old pups’ weights did not vary between groups where orthogonal, deviant and random pups weighed 11.10 ± 0.4, 9.77 ± 0.4 and 10.8 ± 0.2 g respectively. It is worth noting that within the deviant group, male pups (*n* = 5) were significantly heavier than female ones (*n* = 3, *P* < 0.05, Mann–Whitney *U* test) during the first and second week measurements (6.2 ± 0.2, 10.4 ± 0.3 and 4.6 ± 0.1 g, 8.56 ± 0.2 g respectively).

**Figure 3:**
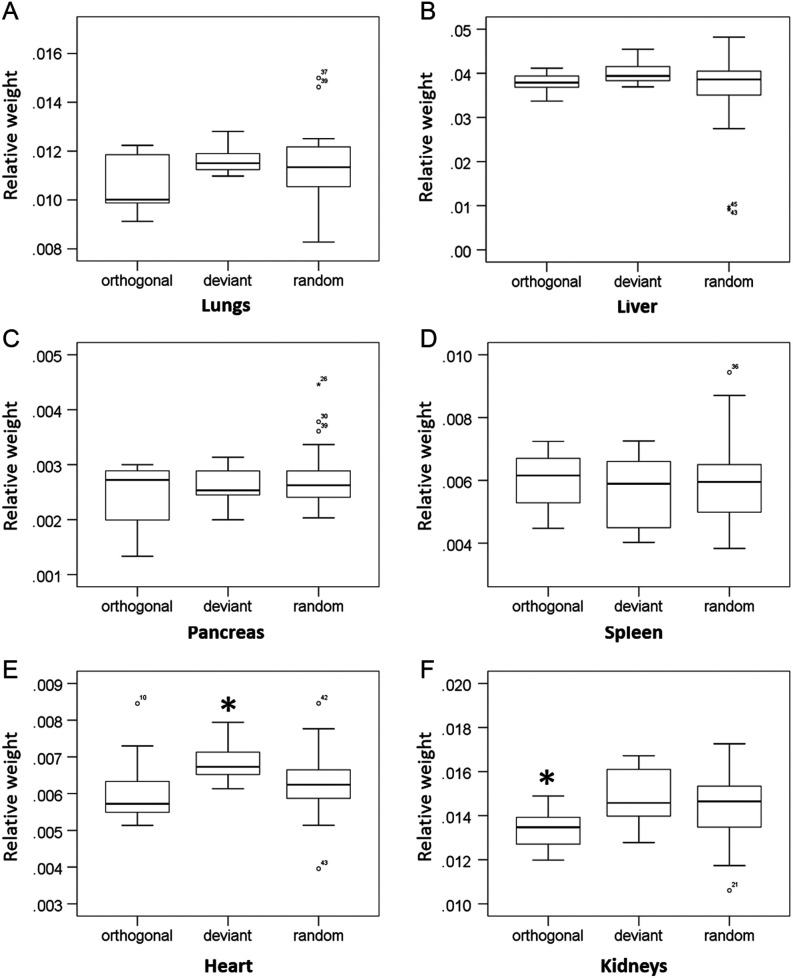
Boxplots comparing the relative organ weights from 2-week-old mice. (A) Lungs, (B) liver, (C) pancreas, (D) spleen, (E) heart and (F) kidneys. Relative organ weights were calculated by dividing the organ weight by the total body weight. A total of 45 pups were analysed: orthogonal (*n* = 12), deviant (*n* = 8) and random (*n* = 25). No significant difference was found among groups for lungs, liver, pancreas and spleen. Heart and kidneys relative weights showed difference among groups (*P* < 0.05 by Kruskal–Wallis test). *Denotes statistical difference (*P* < 0.05 by Mann–Whitney *U* test).

Relative organ weights from 2-week-old mice showed no significant differences (*P* > 0.05) among groups for lungs, liver, pancreas and spleen (Fig. 3). However, a significant difference was found between groups (*P* < 0.05) for heart and kidneys relative weights (Fig. 3). Pups within the deviant group presented significantly heavier relative heart weight when compared with orthogonal (*P* = 0.025) and random (*P* = 0.036) groups. The pups from the orthogonal group presented the lightest relative kidneys weight when compared with deviant (*P* = 0.043) and random (*P* = 0.025) groups. Mann–Whitney *U* test was applied to determine if gender has an effect on relative organ weights within each group. The results showed no significant difference between genders within the three different groups (*P* > 0.05).

## Discussion

In the present study, different cell allocation patterns during preimplantation development were not disturbed by single blastomere removal at 8-cell stage. These patterns might trigger different compensatory mechanism(s) against *in vitro* embryo manipulation, and this may result in short- and long-term consequences for foetus or offspring born after embryo transfer, particularly in deviant embryos. This study raises some questions about the cardiovascular system and kidneys development during foetal development and how this might be affected or predisposed since early preimplantation embryo stages.

Pre-patterning in mouse embryos has been previously reported (Gardner 1997, Piotrowska *et al.* 2001, Piotrowska & Zernicka-Goetz 2002, 2005, Fujimori *et al.* 2003, Plusa *et al.* 2005, Zernicka-Goetz 2005). In the present study, we confirm the presence and a comparable distribution of the three different cell allocation patterns in mouse blastocysts irrespective of mouse strain or embryo cryopreservation at 1-cell stage. These resuls are also in agreement with those previously reported in mouse, bovine and ovine embryos (Hosseini *et al.* 2016, Sepulveda-Rincon *et al.* 2016). Embryo biopsy at the 8-cell stage, whether the biopsied cell is stained or not stained (data not shown), does not seem to alter the cell allocation patterns in mouse embryos similar to what we have previously reported in bovine embryos (Sepulveda-Rincon *et al.* 2016). Assuming that intermingling of cells on the random patterned embryos starts around the fourth cell cycle as in bovine embryos, then these results suggest that cell allocation patterns might be established earlier in development than the 8-cell stage in mammalian embryos. Even though, cell allocation patterns were unaffected by the biopsy procedure, TCC at blastocyst stage were significantly affected in deviant embryos among two different mouse strains and also as previously reported in bovine embryos (Sepulveda-Rincon *et al.* 2016). Hence, cell allocation patterns could be related with different compensatory mechanisms after cell removal. With still an increasing proportion of PGS and PGD procedures and more than ten thousand babies born after embryo biopsy procedures (Peyvandi *et al.* 2011), further understanding of the effects of early embryo micromanipulation and follow-up assessment is needed in order to establish the safety of ARTs in short and long term of development. Therefore, we investigated the effect of cell allocation patterns and its possible impact on further development.

Pregnancy rates in the present study were comparable between groups and in agreement with previously reported studies using NSET device obtaining pregnancy rates higher than 60% (Cui *et al.* 2014). However, there was no statistical difference between groups. It is worth noting that all surrogate females with transferred random blastocysts became pregnant. A possible theory is that random embryos might produce more hyaluronan-promoting cell movements, which is released to the endometrium and cell migration and implantation might be facilitated (Gardner 2015). Also, in the present study, two of the females carrying deviant embryos, presumably committed cannibalism due to problems at delivery or poor offspring health (Wuensch 1993).

Similarly, embryo implantation rates were comparable between the three groups (around 40%) and are similar to those reported after surgical embryo transfer in mice (Hemkemeyer *et al.* 2014). On the other hand, the literature only reports two embryo classifications according to their cell allocation pattern: orthogonal and deviant. The results obtained in the present study are similar reporting no significant differences between groups regarding implantation rates (Alarcón & Marikawa 2003, Liu *et al.* 2012). Nevertheless, our results differ from those in cloned mouse embryos where orthogonal embryos presented higher implantation rates in comparison with deviant ones (Liu *et al.* 2012). Molecular pathways occurring during implantation and crosstalk between embryo and uterus are not clearly understood (Wang & Dey 2006, Cha *et al.* 2012). It is believed that some embryos do not survive embryo transfer procedure due to microenvironment changes and their inability to adapt (Hemkemeyer *et al.* 2014). Moreover, further research is needed in order to determine at which stage the implanted embryos were lost. Pregnancy loss rates in the present study were >50% among groups. Pregnancy loss might be due to aberrant decidualisation; giving rise to placental defects (Chen *et al.* 2015) and therefore affecting foetal growth (Cha *et al.* 2012).

Total body weights at 7 days after birth were different among groups, where the pups from the deviant group presented the lowest weight. While some studies have reported low birth weight after ART when compared with natural conceived babies (Ceelen *et al.* 2009, Bay *et al.* 2014), others have reported only a tendency (Pontesilli *et al.* 2015). In mice, low birth weight has been linked to metabolic syndromes such as type 2 diabetes, obesity and hypertension in adults (Chen *et al.* 2015). Fourteen days after birth, pups among the three groups presented similar total body weights. In humans, it has been reported that babies born with low weight ‘catch up’ within the first 6 months of life (Ceelen *et al.* 2009, Bay *et al.* 2014). Moreover, no differences were found between male and female pups for orthogonal and random groups, only for deviant group. Gender differences have been reported for birth weights in babies after unassisted and assisted conception (O’Neill *et al.* 2014). Further investigations on the long-term health between females and males must be addressed as it has been suggested that mouse male offspring might present a higher risk of developing glucose intolerance after ART (Donjacour *et al.* 2014). In turn, glucose intolerance can develop into cardiovascular diseases, which might affect males or females in a different way (Vlasov & Volkov 2004).

Organ morphometry assessment revealed significant differences among groups for relative weights of heart and kidneys. It is poorly understood why kidneys, blood vessels and the heart are the most affected by early life events (Thornburg 2015). Relative heart weights within the deviant group were higher when compared with other groups. The increase of heart weight has been related with hyperfunction of the myocardium in humans (Vlasov & Volkov 2004) and with an increase on systolic blood pressure and ventricular mass in mice (Donjacour *et al.* 2014). Thus, our results on deviant pups might reflect further consequences on this cardiovascular phenotype and additional investigations could shed some light on how embryo preimplantation development influences the cardiovascular system. Among the long-term effects of ART, cardiovascular problems are the most concerning health issue as an increased risk of developing cardiovascular diseases in comparison with naturally conceived babies has been reported (Padhee *et al.* 2015, Pontesilli *et al.* 2015).

One of the major limitations of this study is the small number of embryo transfers performed. Thus, although the results showed some evidence of effects of preimplantation cell behaviour on subsequent development, further studies are needed to reassure the presented data. Furthermore, it is suggested that a follow-up offspring study is conducted in order to investigate in more depth the consequences of the obtained organ morphometry. Likewise, this work was carried out in mice and caution should be taken when translating the reported results in to humans.

In conclusion, the present study supports the theory that cell allocation patterns during pre-implantation embryo development could be a conserved mechanism in mammalian species at least in two different mouse strains. In addition, a latent concern has been rising about the effects of ART in further offspring health, especially long-term effects. A better understanding on the effects of *in vitro* embryo micromanipulation on further development is paramount not only to improve success rates in ART, but also to ensure the health of resulting offspring.

## Supplementary Material

Supporting Table 1

## Declaration of interest

The authors declare that there is no conflict of interest that could be perceived as prejudicing the impartiality of the research reported.

## Funding

PhD studies of LPSR are funded by CONACyT Mexico.
