# Supplementary material for: Embryo cell allocation patterns are not altered by biopsy but can be linked with further development
Source: Reproduction. 2017 Dec 16;154(6):807–14. doi: 10.1530/REP-17-0514 (PMC5747100; doi:10.1530/REP-17-0514)
Supplement: Supporting Table 1 [file rep-154-807-t001.pdf]

**Supplementary Table 1. Morphokinetic analysis after blastomere removal.** After cell tracing at the two-cell stage, single blastomere removal was performed at the 8-cell stage on the biopsied group, while the control group remained intact. At blastocyst stage embryos were classified into Orthogonal, Deviant and Random groups according to their cell allocation patterns. t8 to t9 interval correspond to the 8- to 9-cell stage during preimplantation, the interval of compaction to cavitation refers to the start of embryo compaction up to the start of cavitation and tB corresponds to the first sign of blastocoel formation. Values are expressed in hours ± SEM (n).

| | | **t8 to t9** | | **Compaction to Cavitation** | | **tB** | |
|---|---|---|---|---|---|---|---|
| **Mouse Strain** | **Group** | Control | Biopsied | Control | Biopsied | Control° | Biopsied⁺ |
| **A** | **Orthogonal** | 9.45±0.49 (16)[a] | 10.54±0.47 (26)[a,b] | 22.59±0.97 (20)[a] | 22.03±0.84 (30)[a,b] | 94.99±1.72 (20)[a] | 96.92±0.87 (30)[a] |
| **A** | **Deviant** | 8.95±0.90 (19)[a] | 8.66±0.47 (33)[a,c] | 21.72±0.50 (20)[a] | 22.01±0.48 (30)[a,c] | 95.31±0.90 (20)[a] | 94.27±0.86 (32)[a] |
| **A** | **Random** | 9.03±0.42 (56)[a] | 9.70±0.25 (80)[a] | 22.28±0.64 (57)[a] | 22.56±0.45 (73)[a] | 95.48±0.68 (59)[a] | 94.53±0.42 (80)[a] |
| **B** | **Orthogonal** | 9.37±0.42 (14)[a] | 10.18±0.65 (13)[a] | 20.94±0.92 (10)[a] | 20.94±0.74 (14)[a] | 72.75±1.66 (16)[a] | 70.05±1.00 (23)[a] |
| **B** | **Deviant** | 7.26±0.80 (14)[a] | 8.52±0.62 (9)[a] | 22.30±1.16 (13)[a] | 22.11±1.44 (13)[a] | 73.76±1.28 (18)[a] | 70.65±1.50 (19)[a] |
| **B** | **Random** | 7.74±0.34 (26)[a] | 8.35±0.64 (25)[a] | 22.35±0.81 (29)[a] | 24.51±1.12 (22)[a] | 72.78±0.83 (35)[a] | 73.11±1.04 (40)[a] |

A: mouse strain B6CBAF1 x B6CBAF1, B: mouse strain B6C3F1 x B6D2F1

°hours post-hCG, ⁺hours post-thawing

Different superscripts denote statistical significance ($p<0.05$) by two-way ANOVA and Bonferroni test between treatment groups and cell allocation patterns within the same mouse strain.
